# Increasing transplantability in Brazil: time to discuss Kidney Paired Donation

**DOI:** 10.1590/2175-8239-JBN-2021-0141

**Published:** 2021-12-17

**Authors:** Juliana Bastos, David José de Barros Machado, Elias David-Neto

**Affiliations:** 1 Santa Casa de Misericórdia de Juiz de Fora, Departamento de Transplante, Juiz de Fora, MG, Brasil.; 2 Hospital das Clínicas da Universidade de São Paulo, Departamento de Transplante, São Paulo, SP, Brasil.

**Keywords:** Kidney Transplantation, Paired Donation, Living Donors, Exchange Donation, Brazil, Transplante de Rim, Doação Pareada, Doadores Vivos, Doação Cruzada, Brasil

## Abstract

**Introduction:**

Kidney transplantation (KT) is the best treatment for chronic kidney disease. In Brazil, there are currently more than 26 thousand patients on the waitlist. Kidney Paired Donation (KPD) offers an incompatible donor-recipient pair the possibility to exchange with another pair in the same situation, it is a strategy to raise the number of KT.

**Discussion:**

KPD ceased being merely an idea over 20 years ago. It currently accounts for 16.2% of living donors KT (LDKT) in the USA and 8% in Europe. The results are similar to other LDKT. It is a promising alternative especially for highly sensitized recipients, who tend to accumulate on the waitlist. KPD is not limited to developed countries, as excellent results were already published in India in 2014. In Guatemala, the first LDKT through KPD was performed in 2011. However, the practice remains limited to isolated cases in Latin America.

**Conclusion:**

KPD programs with different dimensions, acceptance rules and allocation criteria are being developed and expanded worldwide to meet the demands of patients. The rise in transplantability brought about by KPD mostly meets the needs of highly sensitized patients. The Brazilian transplant program is mature enough to accept the challenge of starting its KPD program, intended primarily to benefit patients who have a low probability of receiving a transplant from a deceased donor.

## Resumo

**Introdução:**

O transplante renal (TxR) é sabidamente o melhor tratamento para doença renal crônica. No Brasil, mais de 26 mil pacientes aguardam em lista atualmente. A doação renal pareada (DRP) oferece a um par de doador/receptor incompatível a possibilidade de trocar com outro par na mesma situação, representando uma estratégia para aumentar o número de TxR.

**Discussão:**

A DRP deixou de ser apenas uma ideia há mais de 20 anos. Atualmente é responsável por 16,2% dos TxR com doador vivo (TxRDV) nos EUA e 8% na Europa. Os resultados são semelhantes a outros TxRDV. Essa modalidade representa uma alternativa promissora, especialmente para os receptores hipersensibilizados que tendem a se acumular em lista de espera. A DRP não está limitada a países desenvolvidos. Em 2014, a Índia já publicava excelentes resultados. Na Guatemala, o primeiro TxRDV através de DRP aconteceu em 2011. Porém, a prática permanece limitada a casos isolados na América Latina.

**Conclusão:**

Programas de DRP com diferentes dimensões, regras para aceitação e critérios para alocação estão sendo desenvolvidos e expandidos mundialmente com o objetivo de atender às demandas dos pacientes. O aumento na capacidade de transplantar trazido pela DRP vem ao encontro especialmente das necessidades dos pacientes hipersensibilizados. O programa de TxR brasileiro tem maturidade para assumir o desafio de iniciar o programa de DRP, com o objetivo de beneficiar principalmente seus pacientes que estão em maior desvantagem por apresentarem baixas chances de transplante com doadores falecidos.

## Introduction

Kidney Transplantation (KT) is the best treatment for patients with end stage kidney disease, offering better life expectancy and quality of life to patients^1^. According to the 2020 Brazilian Dialysis Census, an estimated 45 thousand new patients started dialysis in the last year, totaling more than 144 thousand patients undergoing this therapy in the country. The estimated gross mortality of the patients varied between 18 and 20% in period 2016-2019^2^.

The number of KT performed in Brazil is increasing, although it is still less than half of the annual need estimated by the Brazilian Association of Organ Transplantation. Thus, the number of patients on the waitlist grows annually, having surpassed 26 thousand in 2020^3^. In 2003, a study indicated that even if all the deceased patients in the United States donated their organs this would not be enough to meet the backlog demand in that country^4^.

The likelihood to receive a KT becomes even smaller for highly sensitized patients. Before the new allocation policy in the US, the rate of KT with a deceased donor decreased drastically in patients with panel-reactive antibody (PRA) higher than 80% (72% reduction for every 10 points added to the PRA)^5^. These patients displayed 20% higher mortality on the waitlist compared to those with PRA of 0%^6^.

Living donor KT (LDKT), regardless of kinship, offers better patient and graft survival, presenting an alternative to increase the number of organs offered^7^^,^ 8. It is estimated that 35-54% of intended donors fail to donate for immunological reasons (ABO incompatibility or positive crossmatch)^7^^,^ 9. Desensitization protocols, acceptable mismatches and ABO-incompatible transplants have been developed in an attempt to overcome such barriers. However, they are costly and limited to specialized programs^7^^,^ 10. Additionally, those techniques may be associated with higher morbidity and worse long-term results^11^^-^ 13.

Kidney Paired Donation (KPD) offers incompatible donor-recipient pairs the possibility to exchange with another pair in the same situation and performing the transplants, benefiting both recipients^14^. Rapaport described the concept in 1986 and the first procedure was carried out in South Korea in 1991. In South Korea, brain death was not recognized until 1999^15^. KPD programs are a promising strategy to increase the number of high-quality organ transplants and have the added benefit of reaching highly sensitized patients^7^^,^ 13.

## Discussion

Since the 2000s, KPD programs have been expanding globally, and exist in different types and sizes, and as local, regional or national programs^16^. These models have grown more than 200% in the last 10 years in the USA, and account for 16.2% of the LDKT in that country annually^17^^-^ 19.

The first description involved only a simple swap between two incompatible pairs. With experience, different ways to perform the exchanges were developed to optimize the benefits for the recipients enrolled for KPD^16^^,^ 18 ^,^ 20 ^,^ 21 as described below:

- "Closed chains", involving 3 or more pairs, carried out in a way similar to what was described by Rapaport;
- "Endless chains", started by a deceased or non-direct donor and the last living donor donates to a recipient on the waitlist or becomes a link to begin a new chain in the future;
- Exchanges involving a deceased donor, in which the incompatible living donor donates to a listed recipient and, in exchange, the incompatible recipient is prioritized on the waitlist;
- Unbalanced exchanges, when a compatible pair chooses KPD seeking a benefit (higher HLA compatibility, for instance);
- Advanced donation, when there is a chronological incompatibility between donor and recipient, the donation is conducted to a recipient on the waitlist or to start of a new chain. The recipient is given a "voucher" to be prioritized when necessary.

The transplantation capacity of a KPD program depends on the number of pairs registered, on the rate between pairs with ABO incompatibility and positive crossmatch, on the sensitization level of recipients, on the algorithm used for allocation (for example, prioritizing the maximum HLA or chronological compatibility), on the accepted performance models, and on the frequency of match runs for pair allocation^22^^,^ 23.

With that in mind, in many countries, the programs are performed on a regional or national level, mainly favoring highly sensitized recipients who tend to accumulate on the waitlist^24^^,^ 25. To implement more extensive programs, the participating centers must maintain their independence regarding acceptability criteria for their recipients^26^. These criteria can include clinical (such as age or size) and compatibility (degree of HLA compatibility or acceptable mismatches) characteristics.

In 1999, a South Korean study presented their KPD results: patient and graft survival in 5 years were similar to that of haploidentical LDKT and there was no difference in acute rejection^27^.

The first kidney exchange in Europe was conducted in Switzerland in 1999, and 5 years later, the national KPD programs from the Netherlands, the United Kingdom, and Canada had facilitated more than 200 LDKT in each country (29-44% of those registered)^28^. The national Australian program has high rates of highly sensitized recipients registered for KPD (35% of those with 95-100% PRA) and reached the transplantation of approximately 50% of the registered pairs by 2015 (73% of which with PRA 0-50%, 62% with PRA 50-96%, and 25% with PRA >97%)^25^.

In 2016, there were 10 KPD programs in Europe, with different sizes and criteria for acceptance and allocation. Until that year, more than 1300 transplants had been performed in the programs, representing 8% of the LDKT in the continent^29^. Still, in 2016, the first exchange between European countries was of a pair from the Czech Republic that exchanged with an Austrian one, with a cold ischemia time (CIT) of less than 6 hours^30^.

In 2020, 20 years after the first transplant with KPD in the US, the outcomes in up to 7 years of the transplanted recipients were analyzed through the National Kidney Registry (NKR), compared to other LDKT recipients^26^. Those from the NKR had a higher prevalence of African-American patients (18 vs. 13%), PRA >80% (21 vs. 4%), longer dialysis time (1.3 vs. 0.5 years), more patients on public insurance (50 vs. 42%), higher CIT (median of 8.8 h vs. 1 h), higher incidence of delayed graft function (5 vs. 3%), and more patients previously transplanted (25 vs. 12%)^26^. Despite all risk factors, this large registry study that included more than 6 thousand patients, showed that in the first 10 years of the NKR, the outcomes were similar to those recipients of other LDKT^26^. In adjusted analysis, the incidence of graft failure and mortality were similar among the recipients from the NKR and the control groups, with a maximum follow-up of 11 years^26^.

KPD programs are no longer limited to developed countries: in 2017, more than 300 LDKT had been facilitated through KPD in India^31^. A center in the north of that country showed a graft survival of 90.7% after 10 years, with a medium creatinine of 1.3 mg/dL^31^. In the compatible pair subgroup, in which KPD was chosen for a better compatibility, the graft survival was 100% with medium creatinine 1.0 mg/dL.

In 2010, during the Transplantation Bioethics Forum supported by the Latin American and Caribbean Transplantation Society (STALYC), the "Aguascalientes Document'' was drafted recognizing the legitimacy of KPD^32^. In Latin America, the first paired transplant occurred in Guatemala in 2011^33^, and in South America, Argentina was the pioneer in 2015^34^. Nonetheless, in Latin America as a whole, KPD is still limited to a few isolated cases.

One of the main concerns of programs dedicated to creating longer chains is the possibility of withdrawal by the donor after the start of exchanges, as it is difficult to conduct the procedures simultaneously. In 2017, a study assessing such "break" in the chains of KPD was published. In the analysis performed by the NKR on transplantations carried out in the USA between 2008 and 2016, it became evident that the rate of chain breaks was low and mostly due to a medical contraindication on the part of donors^35^. Even when this is the case, the break in the chain does not necessarily mean its end, as a donor undergoes surgery before the intended recipient, necessitating a reassessment of the registry and search for other ways to complete the chain. In that study, the medium size of the chains (number of transplants) that suffered a break did not differ from those completed according to what was previously predicted (4.8 vs. 4.6 exchanges)^( 35)^.

To be ethically justified, the KPD program must consider the 4 principles of biomedical ethics: beneficence, nonmaleficence, justice, and autonomy. Living kidney donation (related or unrelated) is justified when beneficence outweighs nonmaleficence and donor autonomy is preserved. These principles are universal and can be extrapolated to KPD^36^.

Brazilian legislation does not contemplate the possibility of KPD. Law no. 9.434 from February 4, 1997 states that the removal of tissues, organs and body parts of a person in exchancge for payment or promise of reward, as well as for frivolous motives, constitutes a criminal offence^37^. Although organ exchange could be understood as a "promise of reward", it is evident that the law seeks to prohibit the commercialization of organs. In February 2020, a bill was implemented (95/2020) to add to the aforementioned law the following article: "For the effects of this Law, it shall not be considered commercialization the reciprocal donation of organs and tissues (exchange transplantation), so long as it does not involve any monetary benefits stemming from the act"; among other alterations, legitimizing the legality of KPD^38^. Similar legal obstacles have been overcome in other countries to encourage donor exchange^28^. It is important to remember that in KPD, all donors are non-relatives. According to national legislation, they must have prior legal approval, granted by the hospital ethics committee and the organ procurement center.

In 2018, the Brazilian Federal Council of Medicine issued a statement opposing the implementation of KPD in Brazil^39^. The document stated, among other things, that KPD was a controversial concept, still in development and implemented only in a few countries; that it would incur high costs due to the logistical difficulties of the country, with its continental dimensions; that the increase in CIT could affect graft survival; that it would benefit only "a minimal part of the population"; and that it would jeopardize the credibility of the transplant program in Brazil^39^, an analysis that must be re-evaluated in light of currently reported data.

## Conclusion

Globally, KPD ceased being merely an idea over 20 years ago. Programs with different dimensions, rules of acceptance, and criteria for allocation are being developed and expanded, aiming to meet the demands of patients. The rise in transplantability brought about by KPD meets, especially, the needs of highly sensitized patients, with a possibility of combining KPD with desensitization protocols, seeking the best possible result for that currently vulnerable group^24^. There is even a recommendation by American specialists that all centers performing LDKT must join KPD programs, as it is felt that patients would otherwise be disadvantaged^40^.

We believe we have clarified in this review that, contrary to what has been said, KPD programs are no longer "controversial concepts in programs under development"^(39 )^but robust programs that are used almost everywhere in the world and show excellent results, comparable to other LDKT, despite focusing on a population with higher risk and a possible increase in CIT. Another critical issue relates to the main part of the affected population, the highly sensitized people who are sometimes referred to as non-transplantable. A national study in a single center estimated an increase of 7% in the total number of transplants with KPD (which is consistent with the results in the aforementioned countries), and an increase of more than 70% in the number of transplanted recipients with PRA > 80%^41^. If those figures were extrapolated nationally, for example, this would mean an increase of 420 LDKT in 2019.

Thus, there seems to be no reason for Brazil not to join KPD, even if initially only locally and then implemented regionally/nationally according to the acceptance of the centers and the necessary logistical adaptation.

At the HCFMUSP, KPD research seeks to determine the percentage of living donors rejected due to incompatibility and are eligible for KPD and to determine how many recipients would benefit from such a strategy. As part of this program, the first kidney exchange was carried out in Brazil in March 2020 and 28 additional pairs are currently under evaluation.

Nowadays, all the leading countries in world are practicing this procedure and continue to develop it to include more recipients thanks to their excellent results. The importance of this procedure is so great and recognized that in 2012 Alvin Roth and Lloyd Shapley received the Nobel Prize in Economics for their worldwide contribution to the development of algorithms necessary to match a large number of donors and recipients through KDP^42^.

Finally, it is essential to emphasize that KPD also benefits those on the waitlist who do not have a donor, as it reduces the number of recipients waiting for an organ from a deceased donor. We believe that the Brazilian transplant program is mature enough to take up the challenge of starting a KPD program, primarily to benefit patients who have a low probability of receiving a transplant from a deceased donor.
